# Special Issue “Recent Developments in Annexin Biology”

**DOI:** 10.3390/cells9112477

**Published:** 2020-11-14

**Authors:** Ursula Rescher, Volker Gerke, Lina Hsiu Kim Lim, Jyoti K. Jaiswal

**Affiliations:** 1 Institute of Medical Biochemistry, Centre for Molecular Biology of Inflammation, University of Münster, Von-Esmarch-Strasse 56, 48149 Münster, Germany; gerke@uni-muenster.de; 2 Department of Physiology, Yong Loo Lin School of Medicine, National University of Singapore, Singapore 117456, Singapore; linalim@nus.edu.sg; 3 Immunology Program, Life Sciences Institute, National University of Singapore, Singapore 117456, Singapore; 4 Graduate School for Integrative Sciences and Engineering, National University of Singapore, Singapore 119077, Singapore; 5 Center for Genetic Medicine Research, 111 Michigan Av NW, Children’s National Hospital, Washington, DC 20010, USA; 6 Department of Genomics and Precision medicine, George Washington University School of Medicine and Health Sciences, Washington, DC 20010, USA

**Keywords:** Annexin, inflammation, membrane, injury, exocytosis, membrane repair, infection, virus, lipid, cancer

## Abstract

Discovered over 40 years ago, the annexin proteins were found to be a structurally conserved subgroup of Ca^2+^-binding proteins. While the initial research on annexins focused on their signature feature of Ca^2+^-dependent binding to membranes, over the years the biennial Annexin conference series has highlighted additional diversity in the functions attributed to the annexin family of proteins. The roles of these proteins now extend from basic science to biomedical research, and are being translated into the clinic. The research on annexins involves a global network of researchers, and the 10th biennial Annexin conference brought together over 80 researchers from ten European countries, USA, Brazil, Singapore, Japan and Australia for 3 days in September 2019. In this conference, the discussions focused on two distinct themes—the role of annexins in cellular organization and in health and disease. The articles published in this Special Issue cover these two main themes discussed at this conference, offering a glimpse into some of the notable findings in the field of annexin biology.

## 1. Roles of Annexins in Cellular Organization

Several of the studies described in this Special Issue focus on uncovering the cellular roles of annexins, and illustrate the unique involvement of annexins in cellular homeostasis and stress. A homeostatic role of annexin that is examined by Thornton et al. [1] identifies the requirement of intracellular Annexin A2 (AnxA2) in supporting the biogenesis of the Birbeck granule, a specialized compartment found in the Langerhans cells. In contrast, Gabel et al. [2] focus on the extracellular release of AnxA2 by way of secretory granule fusion in the neuroendocrine cells, which results in AnxA2 becoming detectable at the extracellular surface. Matos et al. [3] continue the examination of AnxA2 by focusing on characterizing the mechanism by which it interacts with membranes. They analyze AnxA2 assembly at the membrane by using a novel chemical crosslinker and purified native and mutated AnxA2 proteins to identify how AnxA2 oligomerizes upon binding to membranes with negatively charged phospholipids. Expanding on the lipid and membrane interaction role of AnxA2, Bittel et al. [4] show the importance of the lipid-, protein- and calcium-binding ability of AnxA2 in sensing injury to the plasma membrane, and coordinating a cellular repair response by facilitating injury-triggered vesicle fusion. The role of annexins in membrane repair is also examined by Croissant et al. [5], who make use of correlative light and electron microscopy approaches. Through these studies they identify that plasma membrane injury causes an accumulation of AnxA6 at the site of injury, where it facilitates formation of a plug derived from the membranes at the injury site to help close the hole in the membrane. These roles of annexins in membrane repair, and other roles in structurally remodeling membranes, are reviewed by Bendix et al. [6]. They offer an in-depth perspective on the structural and biochemical roles of annexins through studies conducted at the interface of physics and biology. Transitioning from the structural to a biochemical aspect of annexin biology, Gröper et al. [7] have explored the idea of biased agonism in cell signaling by the cell surface formyl peptide receptors (FPRs), which bind extracellular AnxA1. They describe that the diversity of the homeostatic and inflammatory signals received by FPRs are efficiently distinguished intracellularly not by the selective activation of intracellular signaling pathways, but by way of a source-independent transmission of the danger signal via an agonist bias. Several other studies presented in this Special Issue continue to extend the cellular role of annexins into their implications for health.

## 2. Role of Annexins in Health and Disease

To provide a summary of the involvement of annexins in inflammation and host defense, Dallacasagrande and Hajjar [8] focus on Annexin A2. Their review article describes how AnxA2, which in the homeostatic state sustains anti-inflammatory functions during acute as well as chronic inflammatory states, contributes to disease states when this homeostasis is disrupted. Sanches et al. [9] extend the roles of annexins in inflammation by exploring the involvement of AnxA1 in the inhibition of the release of inflammatory mediators by macrophages. They show that the release of these mediators is increased, leading to greater inflammasome activation in macrophages lacking AnxA1, which causes reduced viability. Hebeda et al. [10] explored the other aspect of AnxA1 in inflammation—acting extracellularly through the FPR-AnxA1 signaling axis. They identify the importance of this process and demonstrate the role of this process in facilitating embryo implantation in the uterus at the blastocyst stage. Vital et al. [11] examined another aspect of FPR-AnxA1—its involvement in thrombus-induced inflammation during sepsis and sickle cell disease. Using intravital imaging together with an AnxA1 mimetic peptide, they observe that targeting this signaling through the Fpr2/ALX receptor helps reduce the severity of the inflammatory response. Needless to state, one of the most pressing health challenges of this past year has been the impact of viral infection on human health. Cui et al. [12] touched upon this topic by exploring the involvement of AnxA1 in host–pathogen interactions in the context of the flu virus. They employed RNA sequencing to examine how the classical autophagy pathway in the host cell is altered by the influenza A virus infection, and discovered the importance of AnxA1 in enhancing autophagy in infected cells. The nexus between annexins, autophagy and human health was further elucidated by Meneses-Salas et al. [13], focusing on the involvement of autophagy in the degradation of AnxA6 and its impact on the devastating childhood disorder Nieman Pick Disease. Finally, two of the studies report on the involvement of annexins in cancer. Focusing on the estrogen receptor-negative breast cancers, Mahdi et al. [14] propose an important role of AnxA2 upregulation in the metastasis and progression of this cancer. This is extended further in the review by Korolkova et al. [15], which takes a broader view of this subject and discusses the contribution of AnxA6 to the progression of various cancers. Focusing on AnxA6, they discuss the relevance of AnxA6 upregulation in the treatment of triple negative breast cancers. These diverse studies offer a bird’s eye view of the several important health and disease-related processes that annexins are implicated in.

## 3. Conclusions

It is apparent that even after 40 years of studying annexins, research on these prolific proteins is still growing. We hope that the work presented in this Special Issue will offer readers a window into the increasing diversity of the cellular and physiological roles attributed to annexins. The biennial Annexin conferences continue to offer a venue for the exchange of these ideas to further our understanding of the roles of annexins, and harness this knowledge to improve the multiple facets of human health that are affected by these proteins.
